# Bind Your Dental Journals

**Published:** 1874-09

**Authors:** 


### BIND YOUR DENTAL JOURNALS.

How many bind and preserve their professional periodicals for ready and easy reference? Observation leads me to say very few. And experience further prompts me to say that it is a great mistake. One does not receive all attainable benefit from one perusal of any lengthy scientific writing; our professional literature included.

Do you consider it a task to read these journals once and not to be thought of for a second time? Then you do not enjoy one of the greatest incentives to a cheerful performance of your every day professional duties. There is nothing more like a sudden meeting with an old and valued friend with whom of all others you crave an interchange of experience in matters of mutual hope and toil, than to run over the well preserved volumes recording the progress of dentistry during the period you have been one of the fraternity, feeling all its deficiencies and rejoicing in all its triumphs.

All know that this is a large item in the list of our profesional enjoyments. And that we should not be deprived of any of them is as true. It is only what we do cheerfully that gives the best results to ourselves and those for whom we labor. All who labor only as necessity drives are slaves to that necessity as much as ever were the subjects of any form of involuntay servitude. It makes a wide difference whether we drive or are driven in the dental practice as in all fields of physical or mental exertion. So, then, let us cultivate a more ready and cheerful acquaintance with the opinions and experiences of others, that by so doing we may be lifted above many of the petty annoyances that might otherwise assume proportions well calculated to lead to discontent with our duties. Therefore preserve your journals for future entertainment and improvement.

To those who have plenty of time and are also liable to wander from their offices in what should be business hours for lack of something to do, I offer some suggestions, not especially scientific, but perhaps quite as profitable and practicable as if they were. By the following way bind your journals at home: Tear off the covers and advertisements. Place a year's numbers, title page and index in place. Take enough book paper for the fly leaves, and fold and lay on the outside pages to keep them clean while handling. Beat the tops and backs even, clamp in a vise very tightly and stitch the backs near the ends with heavy waxed thread such as harness makers use. A stitch at each end with the perforations an inch apart is sufficient. Loop a piece of tape through each of the stitches on each side to paste the "back" to. Compress the volume as firmly as possible in a carpenter's vise with a straight edged board on each side with the straight edge exactly where the leaves must be trimmed to. A sharp drawing knife will shave them off a little at a time as easily as hard wood is cut. Finish with a carpenter's plane and fine sand paper. This trimming I give over to the carpenter only showing them how it is best done. Next cut two pieces of heavy paste board for the boards of the cover or binding, and put them in place on the volume. Spread a heavy piece of colored muslin of the right size on a table and cover with flour paste. Lay the volume and board in the muslin. Remove the volume leaving the boards in position. Turn the muslin over the edges of the boards. Apply more paste where needed and lay the volume in place, carefully adjusting the edges and put in press to dry. Of course, where a good book binder is at hand he may do a little neater work, but no stronger or serviceable.

I like to do any thing I can to draw the general body of the profession into a greater familiarity with its literature, a thing most imperatively needed. So if I can think of some other plan to draw the books through their hands, that possibly they might read them a little more, I shall hardly hesitate to come again.

